# Interventions to Increase Treatment Adherence in Pediatric Atopic Dermatitis: A Systematic Review

**DOI:** 10.3390/jcm4020231

**Published:** 2015-01-27

**Authors:** Alexandria M. Bass, Kathryn L. Anderson, Steven R. Feldman

**Affiliations:** 1 Center for Dermatology Research, Department of Dermatology, Wake Forest School of Medicine, Medical Center Boulevard, Winston-Salem, NC 27157-1071, USA; E-Mails: abass@vcom.vt.edu (A.M.B.); sfeldman@wakehealth.edu (S.R.F.); 2 Department of Pathology, Wake Forest School of Medicine, Winston-Salem, NC 27157-1071, USA; 3 Department of Public Health Sciences, Wake Forest School of Medicine, Winston-Salem, NC 27157-1071, USA

**Keywords:** eczema, atopic dermatitis, atopic eczema, allergy, itch, skin disease, treatment, adherence, non-adherence

## Abstract

Poor adherence to treatment is a major factor limiting treatment outcomes in patients with atopic dermatitis. The purpose of our systematic review is to identify techniques that have been tested to increase treatment adherence in atopic dermatitis. A MEDLINE search was performed for clinical trials focusing on interventions used to increase adherence in atopic dermatitis. Four articles were retrieved. References of these studies were analyzed yielding three more trials. The seven results were evaluated by comparing the intervention used to improve adherence, how adherence was assessed, and the outcome of the intervention tested. Different approaches to increase adherence such as written eczema action plans, educational workshops, extra office visits, and use of an atopic dermatitis educator were evaluated. All interventions increased adherence rates or decreased severity in patients, except for two. The MEDLINE search yielded limited results due to a lack of studies conducted specifically for atopic dermatitis and adherence was measured using different methods making the studies difficult to compare. Interventions including patient education, eczema action plans, and a quick return for a follow-up visit improve adherence, but based on the lack of clinical trials, developing new techniques to improve adherence could be as valuable as developing new treatments.

## 1. Introduction

Atopic dermatitis, like other chronic diseases, requires treatment over an extended period of time making management difficult. Topical medications are the mainstay of treatment and when used as directed, are highly effective [1]. Patients often do not respond as expected, raising the question of nonresponse *versus* non-adherence to the treatment plan [2].

Adherence to treatment of chronic diseases with topical medications is poor. About one-third of patients did not even redeem their prescriptions in a dermatology clinic in Denmark [3]. Even if patients with atopic dermatitis do fill the prescription, adherence may still be poor, with a mean adherence rate of only 40% for five days and 32% for eight weeks [4,5]. Non-adherence may be related to forgetfulness, the time consuming nature of topical treatment, financial burden, lack of a trusting physician-patient relationship, dislike of the prescribed vehicle, steroid phobia or other fears, or a lack of understanding about the disease and proper application of medication [6]. Some 1 to 3.8 billion dollars per year are spent in the United States for treatment of atopic dermatitis; if patients were more adherent, unnecessary return visits and medication changes could be avoided, and healthcare dollars could be saved [7,8].

Studies of interventions to improve adherence have been done in patients with many chronic disorders. Patients with psoriasis had improvement in adherence and outcomes with daily text messages and educational tool interventions [9]. Pharmacist interventions have improved adherence in asthma patients [10]. Positive reinforcements, such as sticker charts, are effective in improving adherence in pediatric patients with chronic diseases such as cystic fibrosis and tuberculosis [11]. The purpose of our systematic review is to identify techniques that have been tested to increase treatment adherence in atopic dermatitis.

## 2. Methods

**Figure 1:**
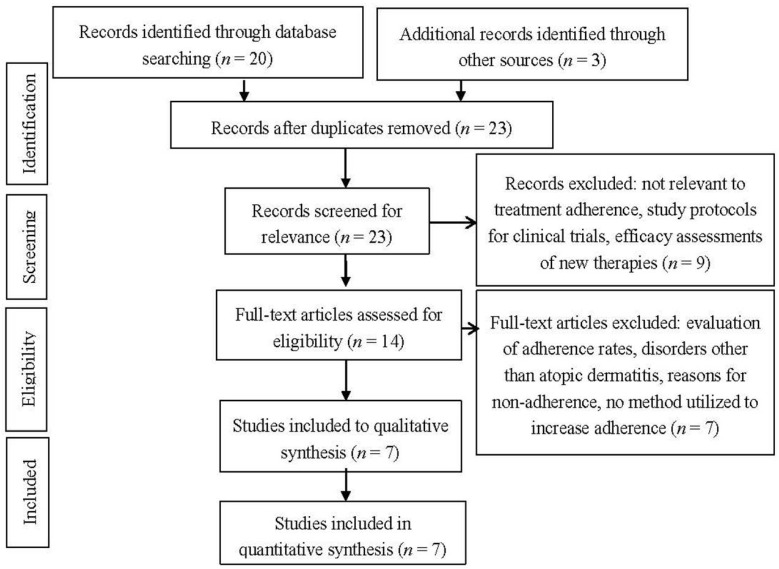
Preferred Reporting Items for Systematic Reviews and Meta-Analyses (PRISMA) 2009 flow chart.

A MEDLINE search was performed containing the keywords “atopic dermatitis” or “eczema” and “adherence” and “treatment” between the dates of 1 January 1984 to 6 September 2014 (Figure 1). The search was limited to clinical trials, full text articles, human subjects, and English language. We only included studies that tested an intervention to improve adherence in atopic dermatitis. Outcomes included were changes in adherence or disease severity. Of the initial 20 results, 4 met criteria for inclusion in the review. References of the identified studies were reviewed for trials meeting the inclusion criteria of our review. One study, located in the references of Moore *et al.* [12], and two studies, located in the references of Shaw *et al.* [13], were found for a total of seven interventions. We evaluated the interventions tested to improve adherence, the method used to assess adherence, and the adherence and/or severity outcomes of the interventions.

## 3. Results

Different methods to increase adherence in atopic dermatitis were evaluated, such as written eczema action plans [14], educational methods [12,13,15,16,17], and extra office visits [18] (Table 1). The studies by Rork *et al.* [14], Moore *et al.* [12], Grillo *et al.* [15], Shaw *et al.* [13], and Chinn *et al.* [17] measured disease severity using the SCORAD (Scoring of Atopic Dermatitis) index and/or quality of life instead of directly measuring adherence. It was assumed that improved severity and/or improved quality of life was caused by increased adherence to the medication.

The study completed by Rork *et al.* used a written eczema action plan (EAP) giving instructions on daily skin care routine and when and where to apply topical steroids, similar to those used in asthma patients. Thirty-five patients participated and were given an EAP at their baseline appointment. The patient’s parents were instructed to complete a survey assessing their child’s disease severity, their treatment comfort level, and whether they had received an EAP previously at baseline and at 3 to 12 months later. At follow-up, 80% of the parents rated their child’s eczema lower on the severity scale, their treatment comfort level increased from 57% to 86%, and 86% found the EAP helpful. Sixty-eight percent of parents attributed the EAP as a factor in improving their child’s eczema [14]. Improved adherence was assumed based on the improved severity.

**Table 1:** Summary of data from seven studies on atopic dermatitis adherence interventions.

Study; Year (From most Effective to Least Effective Intervention)	Adherence Intervention	Sample Size	Measures Used to Assess Adherence and/or Severity	Was Adherence Directly Measured? If So, What Was the Result?	Improvement in Severity	Other Outcomes
Rork *et al.* [14]; 2012	Written eczema action plan (EAP)	35	Parental survey at baseline and at follow up between 3–12 months later addressing severity, treatment comfort level, and if they had received a previous action plan	Not directly measured	80% of the parents rated their child’s eczema lower on the severity scale, 68% attributed improved severity due to the EAP	Parental comfort increased to 86% from 57% at baseline in the intervention group, 86% of parents found the EAP helpful
Moore *et al.* [12]; 2009	Nurse-led eczema workshops	99	SCORAD index	Not directly measured	73% improvement to mild severity in the intervention group *versus* 40% in the control group	N/A
Sagransky *et al.* [18]; 2010	Extra office visit at one week	20	MEMS cap measured adherence, EASI and VAS measured clinical efficacy	Yes by MEMS cap. Mean adherence was 69% in the intervention group *versus* 54% in the control group	Mean improvement between the two groups was not statistically significant. (Improvement in the VAS and EASI scores in the intervention group respectively were 65% and 76% *versus* 36% and 45% in the control group)	N/A
Grillo *et al.* [15]; 2006	Education workshop (2 h session)	61	Severity measured by the SCORAD index, family impact using DFI, and quality of life using the IDQOL and CDLQI	Not directly measured	SCORAD showed mean improvement of 45% at week 4 and 54% at week 12 in the intervention group compared to 7% at week 4 and 16% at week 12 in the control group	DFI, IDQOL, and CDLQI scores showed no significant difference between the groups
Staab, [16]; 2002	Educational program consisting of 6 group sessions of 2 h each	204	Severity measured by the SCORAD index; treatment behavior, dietary restriction, indoor allergen reduction, quality of life, coping, and treatment costs measured by a questionnaire	Yes by survey. After 1 year, 82% of the intervention group *versus* 67% of the control group stated regular use of their skin care products *versus* 88% of the intervention group and 89% of the control group at baseline	Results were not statistically significant. Average decrease in the SCORAD index intervention group was 20 points, compared to 16 points in the control group	Increased dietary restriction, reduction in indoor allergens, increased quality of life, decrease in rumination, and decrease in treatment costs were all seen in the intervention group compared to the control group at 1 year follow up
Shaw *et al.* [13]; 2008	Atopic dermatitis educator (15 min session)	106	Severity measured by the SCORAD index, quality of life using the IDQOL and the CDLQI indices	Not directly measured	Severity decreased 31% in the test group *versus* 21% in the control group but the results were found to be not statistically significant (*p* > 0.05)	No significant difference was noted in the infant’s or children’s quality of life as measured by the IDQOL and CDLQI, respectively
Chinn *et al.* [17]; 2002	Nurse consultation (30 min session)	235	Family impact using the FDI and quality of life using the IDQOL and CDLQI at 4 and 12 weeks	Not directly measured	Not directly measured	Marginal suggestion of benefit in the intervention group using the FDI only at 4 weeks, no significant difference seen between the groups in quality of life

Abbreviations used: SCORAD index (Scoring of Atopic Dermatitis) to measure severity; IDQOL (Infant’s Dermatitis Quality of Life) to measure quality of life in infants; CDLQI (Children’s Dermatology Life Quality Index) to measure quality of life in children; EASI (Eczema and Severity Index) to measure severity; VAS (100 mm Visual Analog Scale) to measure severity by itch intensity; FDI (Family Dermatitis Impact) to measure family impact; MEMS (Medication Event Monitoring Systems) to measure adherence.

In the Moore *et al.* study focusing on eczema workshops, 99 patients with atopic dermatitis either attended a dermatologist-led clinic in which patients were given a plan based on the hospital’s eczema guidelines, the standard protocol, or a nurse-led eczema workshop where in addition to being given a plan, demonstrations of the application process with booklets to take home were also given, the intervention. They were instructed to return for a follow-up appointment in 4 weeks. The disease severity was measured using the SCORAD index which combines objective assessment, disease intensity, a visual analog score of itch, and sleep loss. In this study, 73% of children in the eczema workshop improved to have mild eczema compared to 40% in the control group [12]. Like the study by Rork *et al.* [14], this study did not directly measure adherence, and rather inferred improved adherence in the intervention group based on the improved SCORAD index.

The pilot study performed by Sagransky *et al.* instructed patients with atopic dermatitis to use 0.03% tacrolimus ointment applied to affected areas twice daily and scheduled the patients for a follow-up visit at weeks 1 and 4 in the intervention group *versus* a control group scheduled at only 4 weeks. Adherence was measured by a MEMS (Medication Event Monitoring Systems) cap that recorded the dates and times the tube was opened. Disease was evaluated clinically using the EASI (Eczema Area and Severity Index) and a VAS (100mm visual analog scale) of itch intensity. The adherence in the intervention group ranged from 39% to 114%, with a mean adherence of 69%. In the control group, the mean adherence rate was 54%, ranging from 15% to 79%. There was also clinical improvement in the extra visit group with VAS and EASI scores at 65% and 76%, respectively, compared to 36% and 45% in the control group, however these improvements were not statistically significant (*p* = 0.06 for the difference in EASI outcomes), likely due to the small sample size of only 20 patients [18].

A two hour educational workshop was utilized in the study by Grillo *et al.* to measure its impact on severity, family impact, and quality of life in 61 children with atopic dermatitis without changing their current treatment regimen (exclusion of patients with severe disease taking systemic immunosuppressants). Outcomes were evaluated using the SCORAD index to measure severity, the Dermatitis Family Impact questionnaire to measure family impact, and quality of life by the IDQOL (Infant Dermatitis Quality of Life) and the CDLQI (Children’s Dermatology Life Quality Index) questionnaires at weeks 4 and 12 post-intervention. Mean improvement in severity was 45% at week 4 and 54% at week 12 in the intervention group, compared to 7% at week 4 and 16% at week 12 in the control group. No significant differences were seen in family impact and quality of life amongst the two groups [15]. Improved adherence in the intervention group was assumed based on the improved disease severity.

In 2002, Staab *et al.* tested the effects of a parental training program on the severity, treatment habits, quality of life, and coping strategies of patients with atopic dermatitis measured by the SCORAD index and parent questionnaires. The parental training program consisted of 6 group sessions lasting 2 h each discussing medical, psychological, and nutritional topics as well as sharing personal experience and was compared to a control group at baseline and 1 year. Using the SCORAD index, the change in severity was not statistically significant. Regarding treatment behavior, 82% in the intervention group and 67% in the control group regularly used their skin care products compared to 88% in the intervention group and 89% in the control group at baseline (*p* = 0.041), assessed via questionnaires to the patients’ parents. The intervention group also showed greater improvement in dietary restriction, indoor allergen reduction, quality of life, coping strategies, and treatment costs compared to the control group, evaluated via questionnaires [16].

In the clinical trial by Shaw *et al.*, an educational approach utilizing an atopic dermatitis educator was tested to assess if it would impact the severity, quality of life, and resolution of disease in children. Subjects in the intervention group went to a 15 min education session discussing bathing habits and proper use of emollients at their initial visit after being seen by the dermatologist. There was no significant difference between the control and intervention group in the SCORAD index or infant’s quality of life and children’s quality of life [13]. Adherence was inferred based on the change in the SCORAD index and quality of life measures. Since there was no difference between the control and intervention group, there was assumed to be no difference in adherence.

The study performed by Chinn *et al*. used a single 30 min session with a dermatology nurse demonstrating techniques of applying medication, along with education and advice in the intervention group. Family impact and quality of life was assessed using the Family Dermatitis Index (FDI), the IDQOL, and the CDLQI at 4 and 12 weeks after the intervention, and the changes in these measures was used to infer changes in adherence. Amongst 235 children, only a marginal benefit on family impact was seen at 4 weeks. No significant difference was appreciated in quality of life at 4 or 12 weeks, and therefore there was assumed to be no difference in adherence between the control and intervention groups [17].

## 4. Discussion

One of the most important findings from this systematic review is that few controlled trials on interventions to improve adherence in atopic dermatitis have been performed. Currently, through searching “adherence in atopic dermatitis” on clinicaltrials.gov, only 5 studies are being performed on methods, such as office visits, surveys, and text messages, to improve adherence. For comparison, a MEDLINE search for English, human, clinical trials over the last 30 years (as was performed in the initial search for adherence trials) using the search criteria “atopic dermatitis” and “randomized controlled clinical trial” produced 476 results. Far greater resources are being invested to develop and test new medications for atopic dermatitis, instead of developing methods to improve adherence to treatments that we already know are effective.

Treatment adherence can be challenging for numerous chronic diseases. Many treatments that are expected to be effective do not work because of poor adherence. Interventions that improve adherence may be a better approach than changing what medications are prescribed. Amongst the studies in our review, most of the approaches to increase adherence succeeded with the exception of two. Written eczema action plans, educational workshops, and a shorter time to follow-up were effective methods to increase adherence [12,14,15,16,18]. Due to various measures used in the studies to assess adherence, it is not clear which intervention was the most effective. In the studies of educational interventions—Utilizing an atopic dermatitis educator [13] and a nurse consultation [17]—No significant differences were appreciated between the control *versus* the intervention group. Other studies using educational techniques have improved adherence in chronic diseases and are seen in this study, with the use of workshops and written action plans [12,14,19].

All of the methods investigated involve themes of education, reminders, and frequent communication with patients to augment adherence. The study by Sagransky *et al*. focused on the approach of adding a single early follow up appointment for patients [18]. “White coat adherence” describes the tendency for patients to increase their adherence before follow up appointments and is a common phenomenon in patients with chronic skin diseases treated with topical medications including atopic dermatitis, psoriasis, and acne [4,20,21]. Similar effects are seen with increased flossing that is noticed prior to dental appointments, as well as with increased practicing before piano lessons. If piano teachers behaved like physicians, and instructed children to practice daily for an upcoming recital in eight weeks without weekly piano lessons to provide accountability for practicing, the child would probably begin practicing a few days prior to the recital. The performance perhaps would not be as impressive compared to a child who met with their instructor on a weekly basis, following up on the child and their practice. The same principle can be applied to follow up appointments.

New approaches to improve adherence may be as valuable as efforts to develop new treatments for atopic dermatitis. Different techniques have been proposed to overcome non-adherence in atopic dermatitis, including games, distraction techniques, involving the child in treatment, improving physician-patient relationship, and/or calling and visiting patients within the first week [3,22,23]. However, these methods have not been extensively researched in clinical trials for atopic dermatitis. Even when adherence studies are done, the sample size tends to be small, although a large effect can be seen on adherence and in disease improvement [13,18]. Improving adherence could decrease severity and family burden in patients with atopic dermatitis, as well as provide a more cost effective way to improve outcomes than the development and use of new, expensive agents, decreasing the cost for treatment in atopic dermatitis [7].

The approach of follow-up visits closer to the original appointment is a method lacking clinical research for effectiveness in atopic dermatitis, as it was only tested in the study by Sagransky *et al*. Follow-up with patients, either in person, via phone calls, or via email/text messages, is a successful technique in augmenting adherence in patients with psoriasis [18,20]. In clinical trials of topical medications, follow-up visits are often scheduled at weeks 1, 2, 4, 6, and 8. Trials evaluating the efficacy of topical medications in dermatological conditions such as atopic dermatitis, lichen planus, and genital warts all had patients return for follow-up at variations of 1, 2, 4, and 8 weeks [24,25,26,27]. Even though these visits are occurring to record a patient’s improvement, they are also encouraging good adherence.

The thought of being “checked up on”, especially within the first week of starting treatment, will presumably make patients more apt to pick up their prescription from the pharmacy and begin using their medication. Contact with patients in the beginning of a new regimen may help to ensure they are complying with treatment as directed. It can also be a suitable time to discuss adverse reactions and side effects from the drug that may be affecting the patient. Scheduling a return visit shortly after starting treatment is a strategy to combat fear of potential side effects, especially in mothers of young children with a fear of using topical steroids [2]. Early contact can provide an opportunity for readjustments to the plan if needed.

Other limitations of the review were the studies measured adherence using different methods and some produced subjective conclusions that cannot be duplicated. Five of the seven studies inferred adherence based on changes in disease severity or quality of life [12,13,14,15,16,17]. Staab *et al.* [16] used a questionnaire while only Sagransky *et al.* [18] used objective electronic monitors to measure adherence, a system that is more reliable than self-reported adherence, medication logs, and medication weights [28,29]. Using the same method to measure adherence directly in every study would have provided more confluence amongst our results. However, measuring improvement in disease severity is a useful method to indirectly measure adherence because the ultimate goal of improving adherence is to improve clinical outcomes. Also, the studies did not have patients using the same treatment regimen except for the study by Sagransky *et al.* [18]. Different treatments can be more tedious, leading to differences in efforts required by patients to remain adherent. In addition, the studies only evaluated treatment adherence in pediatric patients, with the target of the intervention being directed towards the parents. It cannot be assumed that the same tactics would produce similar results if directed toward the pediatric patient directly or in adults with atopic dermatitis.

## 5. Conclusions

Effective treatment of atopic dermatitis and other chronic conditions requires adequate long-term adherence to the treatment regimen. Adherence is critically important in treating atopic dermatitis, but few studies have tested ways of improving adherence. Interventions including patient education, eczema action plans, and a shorter time to first follow-up visit appear to improve adherence and treatment outcomes. Given how few clinical trials have been done, developing and testing new techniques to improve adherence may be as fruitful—and perhaps more easily achieved and implemented—as developing new molecules to treat atopic dermatitis.
